# Using Smart Bracelets to Assess Heart Rate Among Students During Physical Education Lessons: Feasibility, Reliability, and Validity Study

**DOI:** 10.2196/17699

**Published:** 2020-08-05

**Authors:** Jiangang Sun, Yang Liu

**Affiliations:** 1 School of Physical Education and Sport Training Shanghai University of Sport Shanghai China; 2 Shanghai Research Center for Physical Fitness and Health of Children and Adolescents Shanghai University of Sport Shanghai China

**Keywords:** physical education, heart rate, validation, feasibility, reliability, Fizzo, Polar, wrist-worn devices, physical education lesson, monitoring

## Abstract

**Background:**

An increasing number of wrist-worn wearables are being examined in the context of health care. However, studies of their use during physical education (PE) lessons remain scarce.

**Objective:**

We aim to examine the reliability and validity of the Fizzo Smart Bracelet (Fizzo) in measuring heart rate (HR) in the laboratory and during PE lessons.

**Methods:**

In Study 1, 11 healthy subjects (median age 22.0 years, IQR 3.75 years) twice completed a test that involved running on a treadmill at 6 km/h for 12 minutes and 12 km/h for 5 minutes. During the test, participants wore two Fizzo devices, one each on their left and right wrists, to measure their HR. At the same time, the Polar Team2 Pro (Polar), which is worn on the chest, was used as the standard. In Study 2, we went to 10 schools and measured the HR of 24 students (median age 14.0 years, IQR 2.0 years) during PE lessons. During the PE lessons, each student wore a Polar device on their chest and a Fizzo on their right wrist to measure HR data. At the end of the PE lessons, the students and their teachers completed a questionnaire where they assessed the feasibility of Fizzo. The measurements taken by the left wrist Fizzo and the right wrist Fizzo were compared to estimate reliability, while the Fizzo measurements were compared to the Polar measurements to estimate validity. To measure reliability, intraclass correlation coefficients (ICC), mean difference (MD), standard error of measurement (SEM), and mean absolute percentage errors (MAPE) were used. To measure validity, ICC, limits of agreement (LOA), and MAPE were calculated and Bland-Altman plots were constructed. Percentage values were used to estimate the feasibility of Fizzo.

**Results:**

The Fizzo showed excellent reliability and validity in the laboratory and moderate validity in a PE lesson setting. In Study 1, reliability was excellent (ICC>0.97; MD<0.7; SEM<0.56; MAPE<1.45%). The validity as determined by comparing the left wrist Fizzo and right wrist Fizzo was excellent (ICC>0.98; MAPE<1.85%). Bland-Altman plots showed a strong correlation between left wrist Fizzo measurements (bias=0.48, LOA=–3.94 to 4.89 beats per minute) and right wrist Fizzo measurements (bias=0.56, LOA=–4.60 to 5.72 beats per minute). In Study 2, the validity of the Fizzo was lower compared to that found in Study 1 but still moderate (ICC>0.70; MAPE<9.0%). The Fizzo showed broader LOA in the Bland-Altman plots during the PE lessons (bias=–2.60, LOA=–38.89 to 33.69 beats per minute). Most participants considered the Fizzo very comfortable and easy to put on. All teachers thought the Fizzo was helpful.

**Conclusions:**

When participants ran on a treadmill in the laboratory, both left and right wrist Fizzo measurements were accurate. The validity of the Fizzo was lower in PE lessons but still reached a moderate level. The Fizzo is feasible for use during PE lessons.

## Introduction

The health benefits of moderate to vigorous intensity physical activity (MVPA) have been documented [1,2]. In China and some European countries, only 20% of children and adolescents achieve the recommended 60 minutes of MVPA daily [3-7]. Physical education (PE) lessons are an important way to promote MVPA in children and adolescents. Such lessons not only provide a chance for students to be active, directly accumulating MVPA over time, but also provide opportunities for students to learn different types of motor or sport skills that may increase their MVPA as well [8,9]. Recognizing the importance of PE lessons for the physical fitness and health of children and adolescents [10], the Centers for Disease Control and Prevention of the United States [11] and Association for Physical Education of the United Kingdom [12] have recommended that children and adolescents engage in MVPA for at least 50% of the total PE lesson time [7]. However, many studies have found that students often do not meet this recommendation [13,14]. In addition, the fact that students cannot reach 50% MVPA in PE lessons can greatly reduce their chance of reaching 60 minutes MVPA daily [15]. Thus, it is important to estimate students’ physical activity during PE lessons and implement interventions to increase students’ participation in MVPA [16].

It is common to evaluate physical activity via HR monitoring, and HR measurements can provide a lot of information about the intensity of physical activity [17]. Monitoring HR can help teachers determine students’ levels of physical activity and improve PE lessons. An increasing number of studies have used objective measurements, such as HR-based devices, to estimate physical activity during PE lessons [18]. The accuracy of these devices has been confirmed [19], but most of them lack feasibility and are too expensive [15]. Therefore, there is a need to identify accurate, inexpensive, convenient, and feasible devices. Increasingly more researchers and consumers are paying attention to wrist-worn wearables [20-22], some of which can be used to evaluate physical activity [23]. However, few studies have examined the use of wrist-worn wearables during PE lessons.

The Fizzo Smart Bracelet (Fizzo) is a wrist-worn wearable made in China, based on photoplethysmography and dependent on optical HR measurement. The Fizzo is not specifically designed to measure physical activity in young children but to measure HR in children, adolescents, and adults. A customized algorithm was designed for the device to measure physical activity based on HR in children and adolescents during PE lessons in school settings. The aims of this paper are the following: (1) to examine the reliability and validity of Fizzo in measuring HR in a laboratory setting; and (2) to examine the feasibility and validity of Fizzo for children and adolescents during PE lessons.

## Methods

### Ethics

The study was approved by the Institutional Review Board of the Shanghai University of Sport (102772019RT034). Before enrollment in this study, written informed consent was provided by every participant.

### Participants

This study was divided into two substudies: Study 1 and Study 2. In Study 1, 11 students from the Shanghai University of Sport, School of Physical Education and Sport Training (Shanghai, China) volunteered to participate. Recruitment was conducted by word of mouth around the university. The participants did not have any musculoskeletal injuries or illnesses.

In Study 2, we randomly selected 10 schools (2 elementary schools, 7 junior high schools, and 1 high school) from Xuhui District in Shanghai, each of which enrolled 2-3 students in PE lessons, for a total of 28 students. At the end of the PE lessons, the students and their teachers (1 teacher/lesson) completed short questionnaires to assess the feasibility of using the Fizzo. Data obtained from the students (Study 1 and Study 2) included age, gender, height, weight, and body mass index (Table 1).

Data were collected in October 2018 (Study 1) and May 2019 (Study 2). The number of participants (n=28) was in line with Wallen et al [24], considering a power of 0.5 and a probability of type I error of 50%. The sample sizes of similar studies ranged from 20 to 60 [21].

### Devices

This study evaluated the Fizzo (The Fifth Zone Fitness Laboratory Company), which is a wrist-worn wearable (cost of ~300¥; US $2.85) that has a battery life of 3 to 8 days. It uses optical sensors to deduce relative volumetric changes in blood perfusion and calculate HR. The Fizzo has a triaxial accelerometer that can measure steps. The data are uploaded to a website [25], where information is stored for up to 90 days, and can be downloaded through an app.

We chose the 2008 Polar Team2 Pro (Polar Electro Oy) as the reference tool in this study. The accuracy of this chest strap has been examined and it is considered to be a standard for the assessment of HR during exercise and training [26,27].

Both devices have a feedback function, but the presentation of data requires a computer or iPad (Apple Inc). In this study, we showed the Fizzo-collected data to the teachers using an iPad.

### Study Procedures and Data Collection

**Figure 1:**
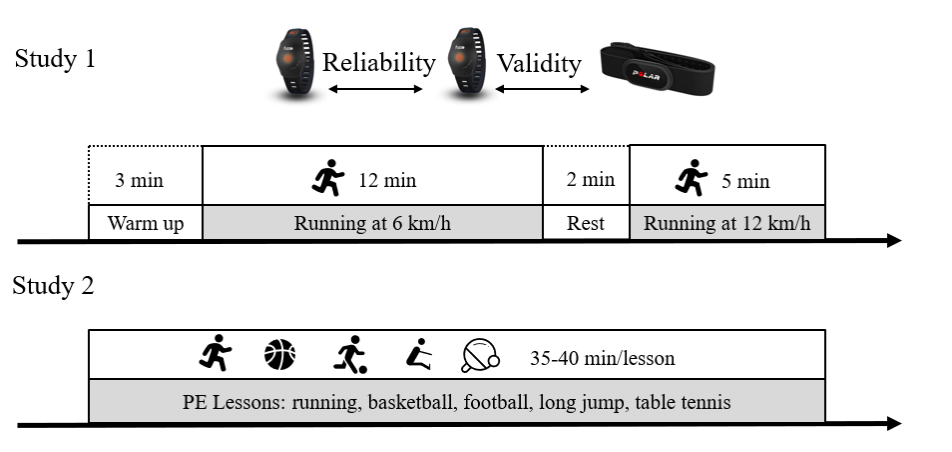
Study 1 and 2 protocol. PE: physical education.

The protocol of Study 1 and 2 are shown in Figure 1. In Study 1, we instructed the students to wear comfortable sportswear and shoes before coming to the laboratory. Before the test, each student put on two Fizzos, one each on their left and right wrists. At the same time, a Polar Team2 Pro (Polar) was placed correctly on the participant’s chest with the help of the researchers and served as the standard. All three devices were tightly secured to ensure skin contact [28]. We chose running speeds of 6 km/h and 12 km/h to correspond with moderate and vigorous intensity movement, respectively. The left Fizzo was compared to the right Fizzo to examine the reliability of the device. Additionally, the left and right Fizzo measurements were compared with the Polar measurements to examine validity. Before the test, participants ran on a treadmill at a self-selected speed for 3 minutes to warm up and to adapt to the environment. Participants ran at speeds of 6 km/h (moderate intensity) and 12 km/h (vigorous intensity) for 12 and 5 minutes, respectively. Between stages, the participants stood for 2 minutes to rest [29]. All participants ran at an incline of zero degrees and all tests took about 22 minutes to complete. Participants could stop at any time if they felt uncomfortable, and every participant successfully finished the test. HR was recorded every second during the test.

In Study 2, we went to 10 schools and measured the HR of 28 students during PE lessons. Before the PE lessons, the students placed a Fizzo on one wrist (according to their own preference; all students chose the right hand) and a Polar band on their chest, which were both secured tightly to ensure skin contact. The start and end time of the lessons depended on the teacher's instructions. When the teachers started the class, we began to measure HR; we stopped when the teachers ended the class. All PE lessons were completed outdoors.

According to studies by Lee et al [17], McNamara et al [30], and Cruz [31], the feasibility of a wrist-worn wearable relies heavily on its accuracy, acceptability, applicability, and usefulness. At the end of the PE lessons, the students and their teachers completed a questionnaire with the help of trained staff. At the same time, we recorded whether or not students removed the Fizzo during the PE lesson. The questionnaire consisted of three questions about the Fizzo that addressed its comfort, application, and helpfulness.

Using a 5-point Likert-type scale, comfort indicators ranged from “very comfortable” to “very uncomfortable,” and the level of difficulty in putting on and removing the Fizzo ranged from “very easy” to “very hard.” The last question asked the PE teachers whether the Fizzo was helpful, with yes/no response options. A total of 28 students and 10 teachers finished the questionnaires. In addition, we invited the teachers to indicate whether or not the Fizzo was helpful for PE lessons. We also asked them to give suggestions, although this was not mandatory. All teachers accepted the invitation.

### Statistical Analysis

The data acquired from the Fizzo and Polar were analyzed using SPSS Statistics (Version 24; IBM Corp). Statistical significance and assumptions for linear statistics were set at *P*≤.05 [32-34]. Before the statistical analyses, all data had been tested for missing values. The data were retained if <10% of the data included missing data points and outliers. In Study 1, there was no missing data, so all data were retained. During PE lessons, 4 students took off their Polar device and the missing data was more than 10% of the total, so their data were deleted. To maintain authenticity, we did not remove outliers, as the Fizzo’s instructions state that HR is accurately measured when placed on either the left or right wrist. Reliability was determined by comparing the left Fizzo data to the right Fizzo data using intraclass correlation coefficients (ICC) for single measurements and absolute agreement from a two-way mixed effect model [35]. ICC shows a measure of relative agreement.

Reliability was determined by calculating the ICC between the two devices with a 95% CI. ICC≥0.9 was considered excellent, 0.90>ICC≥0.75 was considered good, 0.75>ICC≥0.60 was considered moderate, and ICC<0.60 was considered poor [21]. Theoretically, the values of ICC were all positive [36], while the other values were set to zero [37]. Additionally, the reliability between the left Fizzo and right Fizzo was calculated by the mean differences (MDs) and standard error of the mean (SEM) [37,38]. The degree of error can be shown by the MD (SEM) value, with high values indicating high error [34].

To determine validity, ICC with 95% CI for single measurements and absolute agreement from a two-way mixed effect model were used to calculate the relative agreement between the Polar and Fizzo devices in Study 1 and Study 2 [38,39]. In addition, mean absolute percent error (MAPE) was used to assess the degree of error between the standard and the Fizzo, with the equation MAPE = [(Fizzo – Polar)/Polar] × 100%. According to some previous studies [20,40], MAPE≤10% can be considered good, whereas MAPE>10% is considered poor. Finally, the level of agreement was examined using a Bland-Altman analysis and 95% limits of agreement (LOA) between the Polar and Fizzo across the range of HR data (a narrower range is better); this method is recommended to estimate the agreement of medical devices [41].

To determine the feasibility of Fizzo, the number and percentage of “very comfortable” and “comfortable,” “very easy” and “easy,” and “yes” responses were used; a percentage ≥90% was considered good. The number and percentage of students who removed the Fizzo were recorded; a percentage ≤10% was considered good. In addition, the teachers’ views about the Fizzo were analyzed.

## Results

### Overview

The physical characteristics of participants (for both Study 1 and Study 2) are presented in Table 1. There were 11 participants (median age 22.0 years, IQR 3.75 years) in Study 1 (October 2018). Every participant came to the laboratory and was tested twice (on different days, with an interval of 1 week). Finally, we obtained 22 sets of HR data (10 males and 12 females). All participants were right hand dominant.

Data from 4 students were excluded because they removed the Polar chest band during the PE lesson; ultimately, we collected data from 24 students (median age 14.0 years, IQR 2.0 years) in Study 2 (May 2019).

**Table 1:** Participants characteristics for Study 1 and Study 2.

Study and characteristics		Values
**Study 1 (n=11)**		
	Age (years), median (IQR)	22.0 (3.75)
	Males, n (%)	5.0 (45.5)
	Weight (kg), median (IQR)	61.0 (21.9)
	Height (cm), median (IQR)	170.0 (10.8)
	BMI (kg/m^2^), median (IQR)	21.5 (3.8)
**Study 2 (n=24)**		
	Age (years), median (IQR)	14.0 (2.0)
	Males, n (%)	11.0 (45.9)
	Weight (kg), median (IQR)	52.9 (22.5)
	Height (cm), median (IQR)	162.3 (15.6)
	BMI (kg/m^2^), median (IQR)	22.9 (4.3)

### Reliability in Study 1

Table 2 provides the ICC, MD (SEM), and MAPE (SD) for interdevice reliability when running on a treadmill in the laboratory. HR data from the right Fizzo were similar to those from the left Fizzo. The total values for interdevice reliability were evaluated at two running speeds and the devices demonstrated good reliability (ICC=0.99 [95% CI 0.99-0.99]; MD=0.05 [SEM 0.03]; MAPE=1.43% [SD 1.67]). The reliability between the right and left Fizzo at a running speed of 6 km/h was ICC=0.98 (95% CI 0.98-0.98); MD=0.42 (SEM 0.03); MAPE=1.42% (SD 1.64). At a running speed of 12 km/h, reliability values were ICC=0.99 (95% CI 0.99-0.99); MD=–0.66 (SEM 0.056); MAPE=1.44% (SD 1.72). The MD (SEM) at 6 km/h was slightly better than that at 12 km/h; the HR data of the right Fizzo were slightly higher than those of the left Fizzo at 6 km/h and the result was the opposite at 12 km/h. Overall, the reliability between the left and right Fizzo was high (ICC=0.99; MD<0.7; MAPE<2%).

**Table 2:** Interdevice reliability measures of the left versus right Fizzo for heart rate data captured during treadmill running in Study 1.

Characteristics	6 km/h	12 km/h	Total
Intraclass correlation coefficient (95% CI)	0.978 (0.977-0.979)	0.988 (0.988-0.990)	0.990 (0.990-0.991)
Mean difference, right-left (standard error of measurements)	0.42 (0.03)	–0.66 (0.06)	0.05 (0.03)
Mean absolute percentage error, % (SD)	1.42 (1.64)	1.44 (1.72)	1.43 (1.67)

### Validity in Study 1

**Figure 2:**
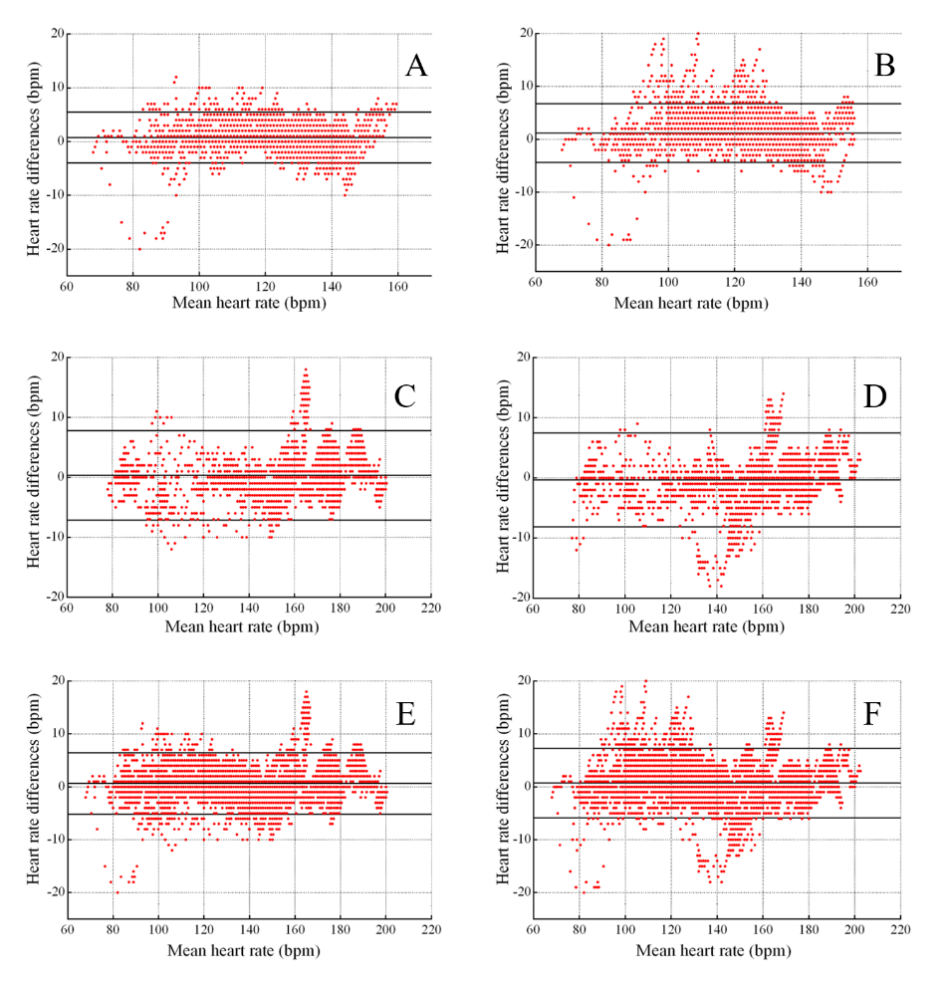
Bland-Altman plot of Fizzo and Polar values for Study 1. A, C, and E are for the left Fizzo and Polar at a running speed of 6 km/h, 12 km/h, and total, respectively. B, D, and F are for the right Fizzo and Polar at a running speed of 6 km/h, 12 km/h, and total, respectively.

The outcomes showed that the mean (SD) HR from the left and right Fizzo were similar to the Polar (Table 3). The left Fizzo (ICC=0.99, 95% CI 0.99-0.99) and right Fizzo (ICC=0.99, 95% CI 0.99-0.99) showed a strong relationship with the Polar device. Additionally, the MAPE (SD) were all small: left Fizzo=1.62% (1.65); right Fizzo=1.82% (2.02). The total validity of both the right and left Fizzo were excellent in the laboratory. The mean difference and agreement between the Fizzo and Polar HR measurements were also shown by the Bland-Altman (Figure 2). Overall, at all running speeds, the left Fizzo had a mean error of 0.64 beats per minute (bpm; lower LOA to upper LOA=–5.18 to 6.45 bpm) and an MAPE (SD) of 1.62% (1.65). The right Fizzo had a mean error of 0.69 bpm (lower LOA to upper LOA=–5.96 to 7.24 bpm) and an MAPE (SD) of 1.82 % (2.02). As the speed increased, mean error changed slightly but the 95% LOA range was larger: left Fizzo 95% LOA=0.78 bpm (lower LOA to upper LOA=–3.95 to 5.52 bpm) and right Fizzo 95% LOA=1.20 bpm (lower LOA to upper LOA=–4.36 to –6.76 bpm) at a speed of 6 km/h; left Fizzo 95% LOA=0.34 bpm (lower LOA to upper LOA=–7.15 to 7.83 bpm) and right Fizzo 95% LOA=–0.32 bpm (lower LOA to upper LOA=–8.13 to 7.48 bpm) at a speed of 12 km/h. The range of HR measurements at 12 km/h was larger than at 6 km/h and the magnitude of the change was small.

At a running speed of 12 km/h, the right Fizzo underestimated HR. Additionally, the left and right Fizzo tended to overestimate HR, and the mean differences were all small.

**Table 3:** Validity of Fizzo versus Polar in Study 1.

Characteristics	6 km/h	12 km/h	Total
Polar, mean (SD)	125.9 (13.8)	158.5 (26.1)	136.8 (24.3)
Left Fizzo, mean (SD)	126.7 (13.3)	158.8 (27.0)	137.4 (24.3)
Right Fizzo, mean (SD)	127.1 (13.3)	158.1 (27.1)	137.4 (24.1)
Left Fizzo, intraclass correlation coefficient (95% CI)	0.984 (0.984 to 0.985)	0.990 (0.989 to 0.990)	0.993 (0.992 to 0.993)
Right Fizzo, intraclass correlation coefficient (95% CI)	0.978 (0.977 to 0.979)	0.989 (0.988 to 0.989)	0.990 (0.990 to 0.991)
Left Fizzo, limits of agreement (lower, upper)	0.78 (–3.95 to 5.52)	0.34 (–7.15 to 7.83)	0.64 (–5.18 to 6.45)
Right Fizzo, limits of agreement (lower, upper)	1.20 (–4.36 to 6.67)	–0.32 (–8.13 to 7.48)	0.69 (–5.96 to 7.24)
Left Fizzo, MAPE^a^ (SD) (%)	1.56 (1.52)	1.74 (1.88)	1.62 (1.65)
Right Fizzo, MAPE (SD) (%)	1.80 (2.05)	1.85 (1.95)	1.82 (2.02)

^a^MAPE: mean absolute percentage error.

### Validity in Study 2

The contents of the PE lessons included running, basketball, football, long jump, and table tennis. Across the PE lessons, the mean (SD) HR between the Fizzo and Polar devices were similar: 137.6 bpm (26.8 bpm) and 140.2 bpm (24.7 bpm), respectively (Table 4). Bland-Altman analysis showed that the Fizzo had a mean error of –2.60 bpm, while the 95% LOA between the two devices ranged from –38.89 to –33.69 bpm. The ICC between the Fizzo and Polar devices was 0.742 lower than in the laboratory. The MAPE was 8.89% for the PE lessons, higher than in the laboratory (1.82%). The Fizzo slightly underestimated HR compared to the Polar during PE lessons.

**Table 4:** Validity of Fizzo versus Polar in Study 2.

Characteristics	Value
Polar, mean (SD)	140.2 (24.7)
Fizzo, mean (SD)	137.6 (26.8)
Fizzo versus Polar, intraclass correlation coefficient (95% CI)	0.742 (0.739 to 0.746)
Fizzo versus Polar, limits of agreement (lower, upper)	–2.60 (–38.89 to 33.69)
Fizzo versus Polar, mean average percentage error, % (SD)	8.89 (11.04)

**Figure 3:**
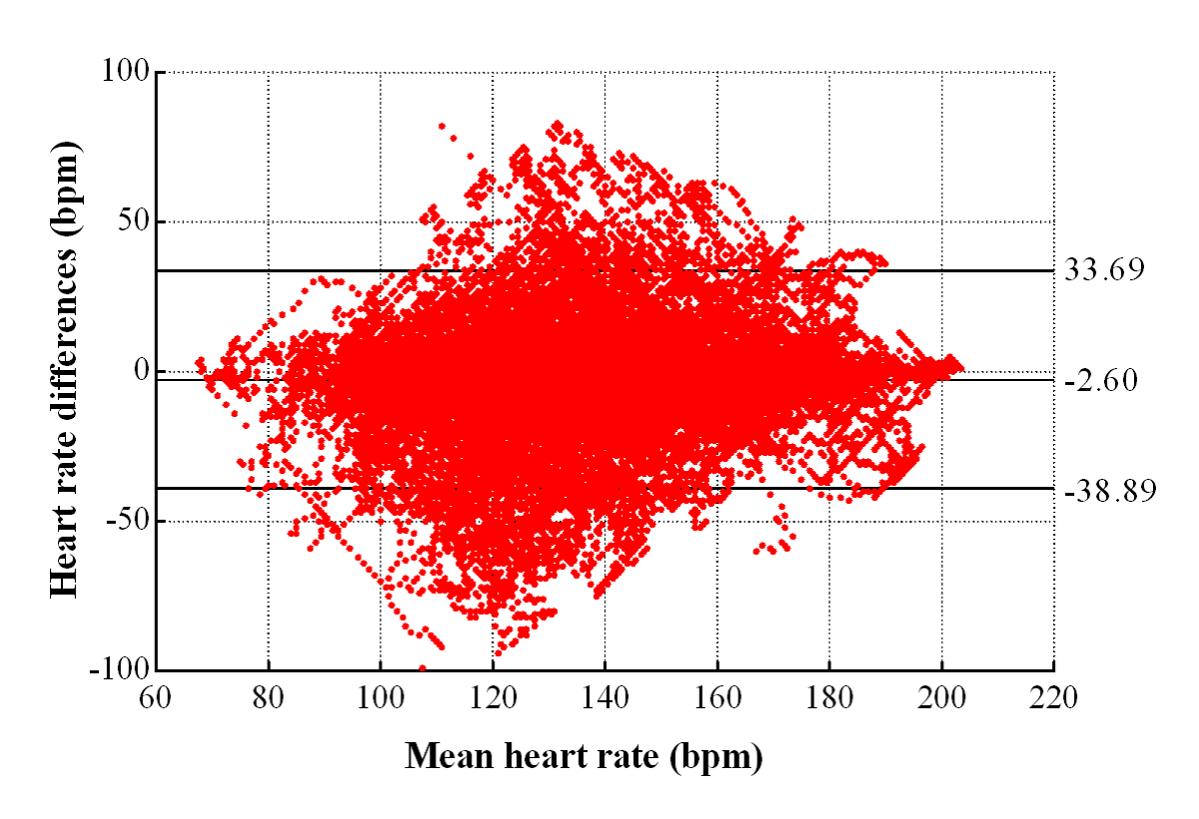
Bland-Altman plot of Fizzo and Polar values in Study 2.

The range of the LOA was greater than in the laboratory. The HR data had a mean error of –2.60 bpm (lower LOA to upper LOA=–38.89 to 33.69 bpm). The LOA of the Bland-Altman plots are presented in Figure 3. The Fizzo had the narrowest LOA in the laboratory condition, and broader LOA in the PE lesson condition. The ICC was lower during the PE lessons (0.748) than in the laboratory (>0.99). The MAPE was larger for the PE lessons (8.89%) than in the laboratory (1.82%). The validity of Fizzo in the laboratory is better than in the PE lessons.

### Feasibility in Study 2

The feasibility responses from students and teachers were highly consistent (Table 5). All students chose “very easy,” and 71% of students chose “very comfortable” while 29% of students chose “comfortable”; no one chose the “neutral,” “uncomfortable,” or “very uncomfortable” options. The comfort level of Fizzo is high; none of the students felt uncomfortable during the PE lessons. Regarding application, all students chose “very easy,” which means that Fizzo is convenient to wear and remove, and it is easy to put on for PE lessons. All teachers chose “yes” for “helpful”; they believed that the Fizzo has practical value and it is useful for PE lessons. They think that the Fizzo helps them track the students’ physical activity levels more conveniently and helps them create more reasonable course content. Furthermore, they think that the Fizzo is very suitable for PE lessons. All the teachers were interested in using the Fizzo in the future. No students tried to remove the Fizzo during the PE lessons. The results showed that it is feasible to use the Fizzo during PE lessons.

**Table 5:** Questionnaire results for feasibility.

Questionnaire responses		Students (n=28), n (%)	Teachers (n=10), n (%)
**Comfort, n (%)**			
	Very comfortable	20 (71)	—^a^
	Comfortable	8 (29)	—
	Neutral	0 (0)	—
	Uncomfortable	0 (0)	—
	Very uncomfortable	0 (0)	—
**Ease of application and removal, n (%)**			
	Very easy	28 (100)	—
	Easy	0 (0)	—
	Neutral	0 (0)	—
	Hard	0 (0)	—
	Very hard	0 (0)	—
**Helpful, n (%)**			
	Yes	—	10 (100)
	No	—	0 (0)

^a^Not applicable.

## Discussion

### Principal Findings

The results indicated that the validity and reliability of the Fizzo were excellent in the laboratory. The validity of the Fizzo in the PE lessons was lower than in the laboratory setting, but still moderate. The Fizzo was feasible for PE lessons.

The intensity, duration, and frequency of physical activity can be inferred through HR. Measuring the HR of students accurately and conveniently has great benefit when aiming to increase MVPA to achieve better health preservation effects. The emergence of wrist-worn wearables provides a realistic basis for the achievement of these goals [42]. However, there are still few studies assessing the validity and feasibility of wrist-worn wearables during PE lessons. The purpose of this study was to examine the reliability and validity of a new wrist-worn wearable, Fizzo, in the laboratory and during PE lessons. 

Some studies have stated that trackers’ reliability and validity may be affected by which wrist the device is worn on [43,44], as there may be some differences in reliability or validity between the right and left wrist (dominant and nondominant). However, in Study 1, we found that validity and reliability were not affected by which wrist the Fizzo was worn on; the HR values as measured by the left and right Fizzo were similar to the HR values obtained from the Polar device. The validity and reliability of Fizzo were almost unaffected by running speed, and Fizzo maintained its high performance when the running speed was increased. This finding is different from previous studies, which found that accuracy became worse when the running or jogging intensity was increased [16]. The stronger relationship between the Polar and Fizzo devices during the laboratory study can be attributed to more stable experimental controls. Furthermore, when running on a treadmill, the movements of the left and right arms were similar and were not affected by external forces, which may be another reason for this differing result [45].

In Study 2, the validity of the Fizzo was lower during the PE lessons compared to the laboratory, but still showed moderate accuracy (ICC=0.742; mean error=–2.60 bpm, LOA=–38.89 to 33.69; MAPE=8.89%). ICC was very close to the “good” level (0.75); increasing the sample size may impact this result. The MAPE and the range of LOA during PE lessons were larger than in the laboratory and the Fizzo tends to underestimate HR during PE lessons. The Fizzo was easy to use and comfortable for students. Furthermore, it was helpful for teachers during PE lessons and the teachers were interested in using it in the future, which demonstrated the feasibility of the use of the Fizzo. As indicated by the different performance levels of the Fizzo between Study 1 and 2, the validity of the Fizzo may be not influenced by physical activity intensity (running speed) but may be affected by sex, environment, and the type of physical activity [45]. In PE lessons, the students’ arms, where the Fizzo was worn, were often subjected to external forces, such as slapping a basketball. These conditions may cause the device to lose skin contact and leak light, resulting in measurement errors. It is also possible that sweat between the device and the skin may cause errors. Compared to Study 1, it was not only the type of exercise that changed in Study 2, but also the environment; these changes may have caused the reduced validity. In the laboratory, the environmental light was relatively stable, but in the PE lessons, it was always changing. The Fizzo uses an optical sensor to measure HR, so it is very sensitive to light. If the Fizzo is not worn tight enough, changes in light may affect its accuracy.

With the development of new technologies, wrist-worn wearables for measuring HR will be an alternative to the chest strap [46]. Wearing the Fizzo on the wrist was more convenient and easier to use than the chest strap; all students considered the Fizzo as comfortable, as well as easy to wear and take off. A chest-worn device is more troublesome, and clothes must be taken off. In addition, it needs to be wiped with alcohol, so it is inconvenient, particularly when the weather is cold. The break between different classes is about 10 minutes in primary and secondary school in China; before the PE lessons started, there were only one or two students who had enough time to apply the Polar bands with the help of assistants. It is very difficult to apply the chest band by oneself. The content of the PE lessons is heterogeneous and complicated and could cause chest-worn devices to fall off, which was why we went to 10 schools but only got 24 samples. Conversely, the Fizzo is worn on the wrist and was less restrictive during physical activity compared with the Polar; the wrist-worn wearable is more comfortable and more acceptable. The teachers considered it helpful for monitoring the physical activity level of their students. According to the HR values of the students, the teachers could choose more reasonable physical activity intensity levels. All teachers believed the Fizzo has practical value and the device showed high feasibility.

When conducting a long-term physical activity surveillance study, a valid survey is usually defined as >10 hours of wear time every day for at least 4 days [47]. The wrist-worn wearables may be more convenient than the chest bands and devices in other positions [48]. Therefore, we need to pay attention not only to accuracy but also to the adherence of participants. Although the Fizzo performed well in PE lessons, further studies are required to prove it can be used in daily life.

To avoid the social desirability response bias with objective measures of physical activity [48], both the Polar and Fizzo do not have a display. However, sometimes feedback is needed; the Fizzo can show almost all students’ data (60 students) at the same time on a smartphone or an iPad, while the Polar can show data from 15 students on a computer. Some devices can receive and show the data up to a distance of 200 meters, but sometimes the scope of activities of students in PE lessons far exceeds this distance. The Fizzo can cover the entire playground if small antennas are put near the perimeter of the venue. The Fizzo is more feasible for the monitoring of PE lessons.

Like Polar, most devices for the collection of physical activity data are too expensive; the price of the Polar device is quite high (about 100,000¥ [US $948.7] for 15 Polar bands and a computer). The price of 15 Fizzo is significantly lower (about 4500¥ [US $42.75]), and the computer or iPad can be purchased by oneself, so it is more affordable for researchers. When research funding is limited, researchers can choose cheaper equipment. The Fizzo may be a good choice as it has good validity, reliability, and feasibility during PE lessons. Although many types of PE classes are not included in this study, the available results indicate that the Fizzo has a relatively large application potential in a PE class setting.

### Limitations

This study has limitations. The participants were healthy students, while subgroups with known arrhythmia and many types of PE lessons were not included in this study; thus, we cannot be sure of the accuracy of the Fizzo in other populations, including people with heart disease. The small sample size may have affected the results of Study 2, which may limit its generalizability.

To match the end of the Fizzo measurements with the end of the Polar measurements, we only measured HR while participants were running; we did not measure HR after the participant stopped running.

Students were of different ages and included elementary, secondary, and high school students; there may be some reliability and validity differences among these three groups during PE lessons, but that was not distinguished in this study. Physical activity types may have an impact on the reliability and validity of the device, but our study did not classify different types of physical activity; therefore, we cannot be sure which types of physical activity were causing the changes in reliability and validity.

### Conclusions

This study shows that the Fizzo has good reliability and validity during moderate and vigorous intensity running on a treadmill in the laboratory. Compared to the laboratory results, the validity of the Fizzo was decreased in PE lessons but still reached a moderate level. The main factor affecting device reliability and validity may not be the intensity of physical activity but the type of physical activity. More research is needed to determine which types of physical activity affected the reliability and validity. Ultimately, the Fizzo is accurate, comfortable to wear, easy to apply and remove, and has a high application value in a PE lesson setting.

## Abbreviations

bpm: beats per minute
Fizzo: Fizzo Smart Bracelet
HR: heart rate
ICC: intraclass correlation coefficient
LOA: limits of agreement
MAPE: mean absolute percentage error
MD: mean difference
MVPA: moderate to vigorous intensity physical activity
PE: physical education
Polar: Polar Team2 Pro
